# Improving Empathy in Healthcare Consultations—a Secondary Analysis of Interventions

**DOI:** 10.1007/s11606-020-05994-w

**Published:** 2020-07-14

**Authors:** Kirsten A. Smith, Felicity L. Bishop, Hajira Dambha-Miller, Mohana Ratnapalan, Emily Lyness, Jane Vennik, Stephanie Hughes, Jennifer Bostock, Leanne Morrison, Christian Mallen, Lucy Yardley, Hazel Everitt, Paul Little, Jeremy Howick

**Affiliations:** 1 University of Southampton , Southampton, UK; 2 Keele University , Keele, UK; 3 University of Bristol , Bristol, UK; 4 University of Oxford , Oxford, UK

**Keywords:** empathy, consultation, communication

## Abstract

A recent systematic review of randomised trials suggested that empathic communication improves patient health outcomes. However, the methods for training healthcare practitioners (medical professionals; HCPs) in empathy and the empathic behaviours demonstrated within the trials were heterogeneous, making the evidence difficult to implement in routine clinical practice. In this secondary analysis of seven trials in the review, we aimed to identify (1) the methods used to train HCPs, (2) the empathy behaviours they were trained to perform and (3) behaviour change techniques (BCTs) used to encourage the adoption of those behaviours. This detailed understanding of interventions is necessary to inform implementation in clinical practice. We conducted a content analysis of intervention descriptions, using an inductive approach to identify training methods and empathy behaviours and a deductive approach to describe the BCTs used. The most commonly used methods to train HCPs to enhance empathy were face-to-face training (*n* = 5), role-playing (*n* = 3) and videos (self or model; *n* = 3). Duration of training was varied, with both long and short training having high effect sizes. The most frequently targeted empathy behaviours were providing explanations of treatment (*n* = 5), providing non-specific empathic responses (e.g. expressing understanding) and displaying a friendly manner and using non-verbal behaviours (e.g. nodding, leaning forward, *n* = 4). The BCT most used to encourage HCPs to adopt empathy behaviours was “Instruction on how to perform behaviour” (e.g. a video demonstration, *n* = 5), followed by “Credible source” (e.g. delivered by a psychologist, *n* = 4) and “Behavioural practice” (*n* = 3 e.g. role-playing). We compared the effect sizes of studies but could not extrapolate meaningful conclusions due to high levels of variation in training methods, empathy skills and BCTs. Moreover, the methods used to train HCPs were often poorly described which limits study replication and clinical implementation. This analysis of empathy training can inform future research, intervention reporting standards and clinical practice.

## INTRODUCTION

Empathy is defined in numerous ways; however, in healthcare, there is emerging consensus that it involves therapeutic empathy, whereby a HCP (‘Healthcare Practitioner’: a medical professional, such as a nurse or surgeon) puts themselves in a patient’s position to acknowledge their feelings, concerns and expectations and behaves in a way to show that they understand.^1,2^ These behaviours could be using verbal or non-verbal behaviours to convey empathic affect (e.g. saying ‘I understand how you are feeling’ or using eye contact) or behaviours that encourage empathic healthcare interactions so that the patient feels listened to and supported (e.g. explaining rationale for treatment, checking the patient understands). Understanding and compassion in healthcare is of global importance: the World Health Organization has identified person-centred care as a crucial component of healthcare, “measurably improving the quality of care, the success of treatment and the quality of life of those benefiting from such care” (^3^ P46). In the United Kingdom (UK), healthcare policy increasingly emphasizes the importance of “compassion, dignity and respect” in patient interactions.^4^ This is relevant and timely as national data reports empathy levels among HCPs are decreasing.^5^ In UK primary care, where over ten million HCP contacts occur annually, patient satisfaction reached an all-time low at 65% in 2017.^6^ These findings are concerning given evidence that low empathy strongly correlates to low satisfaction and increased levels of anxiety, distress and pain.^1^ Patient experiences of empathy have additionally been linked to other health outcomes including blood pressure, all-cause mortality and faster resolutions of self-limiting illness.^1,7,8^ Moreover, empathy has been shown to be beneficial to HCPs in reducing stress and burnout.^9^

While the benefits of empathic communication are broadly accepted, evidence of ongoing patient dissatisfaction with healthcare consultations^6^ and the decline in HCP empathy over time^5^ suggests that more needs to be done to translate this evidence and implement it in practice. A problem with the current literature is that there is no agreed method of training empathy, or consistent content to such training. A detailed description of the methods used to encourage empathic care could therefore move this field forward by making the evidence implementable. To achieve this, we selected Howick et al.^10^ as the basis for our analysis as it provides recent high-quality RCT data with physical or psychological outcomes for the patient’s health. It also details only qualified HCP training, which has substantial differences to student training in time invested and setting. We aimed to extract the core details of the HCP empathy training from each included study to better understand these interventions and inform the development of successful implementable evidence-based empathy training for HCPs.

### Aims

1. To investigate the components of effective training for HCPs in empathic communication. This involved identifying the:
  - Methods used to train HCPs,
  - Empathy behaviours that HCPs were trained in and
  - Behaviour change techniques (BCTs) used to train HCPs.
2. To investigate which empathic behaviours are most effective for improving patient outcomes.

## METHODS

### Sampling

Howick et al.’s systematic review^10^ contained seven randomised trials that compared outcomes in patients who had been treated by (a) HCPs trained to be more empathic and (b) HCPs who had not been trained. The review excluded non-randomised trials, and any study that did not compare the (downstream) effect on patients. Trials that measured change in practitioner empathic behaviour, but not patient outcomes, were excluded. The conditions and experiences included in these studies were chronic pain,^11–13^ anxiety,^14^ distress among cancer patients,^15^ irritable bowel syndrome^16^ and satisfaction after primary care consultations.^17^ The average effect size was modest (SMD − 0.18 [95% CI, − 0.32 to − 0.03]), and study heterogeneity was medium (*I*^2^ = 55%). Topic experts were consulted to help identify additional papers, and a rapid search for more recent research matching Howick et al.’s inclusion criteria found no additional randomised trials (November 2018).

### Analyses

We used a qualitative content analysis approach^18,19^ to describe and analyse the methods used to train HCPs in empathic communication, and the specific behaviours that the training intended to encourage. In this approach, text is searched for certain types of content, which is then extracted, categorized and summated. This approach was chosen as it allowed us to condense the data and potentially evaluate which empathy behaviours and empathy training methods are most effective.

First, papers were read in detail and systematically searched for all content about the training, which was then extracted into a spreadsheet (by JH and KS). An inductive approach was used to code (1) methods used to train HCPs, including the duration and deliverer of the training, and (2) empathy behaviours that the HCPs were trained in.

Interventions use many different approaches that aim to change a person’s behaviour, often involving many complex components. The Behaviour Change Technique Taxonomy was developed as a tool to extract the active ingredients in different interventions so that they could be replicated, synthesized and implemented. It covers typical teaching techniques like demonstration, but also other techniques to change behaviour, such as different types of reward, social support, feedback and habit formation. We are interested in not only how and what the HCPs were taught, but what was done to motivate them to implement and sustain the empathic behaviours.

A deductive approach was used to code the BCTs used to train HCPs. This involved reviewing descriptions of training to identify any use of the 93 BCTs defined and described in an established BCT taxonomy.^20^ Finally, papers were evaluated according to whether they reported using each training method, empathy behaviour and BCT identified. At least two authors experienced in qualitative analysis (FB and KS) checked the coding for each of (a) empathy training methods, (b) empathy training content and (c) BCTs.

Effect sizes reported in Howick et al.^10^ were then compared across components to explore qualitatively if there were any differences in training components between highly effective and less effective interventions.

## RESULTS

### Empathy Training Methods

Full details and characteristics of the included studies are presented elsewhere (Howick et al.). Table 1 presents a summary of the HCP training methods extracted from the papers included in the systematic review. All interventions took place at the HCPs’ place of work, including primary and secondary care settings.

**Table 1:** Methods of HCP Training Extracted Using Content Analysis

Study	Country	Domain	Intervention	Trainee	Training deliverer	Training duration	Components
Chassany 2006	France	Pain management in osteoarthritis of knee or hip	Intervention delivered by empathy-trained doctor vs. consultation delivered by untrained doctor	GP	Facilitator and expert	4 h	Videos of consultations Peer discussion Self-experimentation Creation of recommendation list Handout for patients Eight reminders post-training
Fujimori 2014	Japan	Breaking bad news to cancer patients	Intervention delivered by empathy-trained oncologist vs. treatment delivered by untrained oncologist	Oncologist	Psychiatrists, psychologists and oncologists	10 h	Lecture (evidence of patient preferences, instructions) Videos of consultations Role-playing Peer feedback Peer discussion
Kaptchuk 2008	USA	Sham acupuncture for irritable bowel syndrome (IBS)	Augmented consultation with acupuncturist vs. time-limited patient-practitioner relationship (initial consultation duration < 5 min)	Acupuncturist	Unknown	20 h	Training manual Video of consultations Role-playing Recording self Consultation feedback post-training
Little 2015	UK	General practice doctor consultation with adults	Intervention delivered by empathy-trained doctor vs. treatment delivered by untrained doctor	GP	Medical student	5-10 min for training, then up to 2 h for self-recording/monitoring	Brief one-to-one training (evidence of patient preferences, instructions, goal setting, action planning) Recording self Self-monitoring Summary sheet
Soltner 2011	France	Preoperative anaesthetist visit for gynaecological problem requiring day-care procedure	Consultation by HCP trained to provide additional empathy (with 5 min extra time) vs. consultation delivered by HCP instructed to give a neutral consultation.	Anaesthetist	Unknown	Unknown	Role-playing Recording self (as part of calibration)
Vangronsveld 2012	Sweden	Interview with nursing staff about their back pain	Interviewer actively/empathically listening and validating during a 15-min interview vs. non-validating interview	Interviewer with psychological background	Two trained therapists	Unknown	Interview scripts
White 2012	UK	Real/placebo acupuncture for back pain	Empathetic consultation vs. non-empathetic consultation	Physiotherapist, nurse and licenced acupuncturist	Unknown	Unknown	Social support from other trainees post-training

We found that the core methods of training were:

- Face-to-face training (*n* = 5)
- Role-playing (*n* = 3)
- Videos of model consultations (n = 3)
- Videos of self in consultation (n = 3)
- Post-training material (hand-outs, reminders, feedback, meetings) (*n* = 4)
- Presentation/talk (*n* = 2)
- Discussion with peers (*n* = 2)

Training could include multiple methods—see Table 1 for the components described in each paper.

Training duration varied from 2 to 20 h. Three studies described training HCPs in groups of 4–36. Five studies described using face-to-face training. The types of trainer used were HCPs (e.g. psychiatrists, psychologists, oncologists, therapists) and a medical student. Role-playing was used for three of the studies. Videos were used extensively; three studies used videos of others as part of the training, and three studies required the HCPs to have videos made of themselves. Two studies described a lecture or talk being given to HCPs prior as part of their training. Two studies used discussion with other HCPs. Four studies described content or contact provided after the initial training session: reminders, feedback on their videos, a summary sheet and regular meetings with other trained HCPs. One study provided materials (an informational leaflet) to give to patients.

### Empathy Training Content

**Figure 1:**
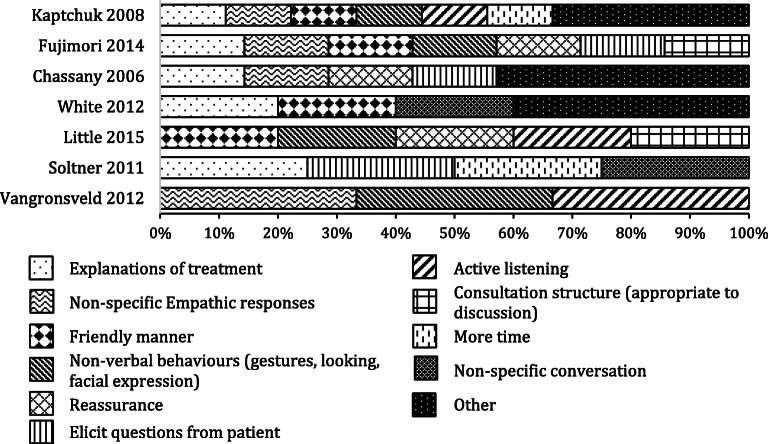
**Overview of training content in each study. ‘Other’ includes all content only reported in a single study; see Table** **2****for details.**

Figure 1 summarises the different empathy behaviours that the HCPs were trained in. Between 3 and 9 (median = 7) empathy behaviours were identified in each reported training (see Table 2). The most common element that the HCPs were trained in was providing explanations of treatment (*n* = 5). Providing non-specific empathic responses (e.g. “I show my patient that I believe his/her pain is genuine”^11^), a friendly manner (e.g. being friendly, warm or cordial to the patient) and non-verbal behaviours (e.g. nodding, leaning forward) were also popular (*n* = 4 each), followed by active listening (using body language and short responses like “hmm, ok” to show you are listening), eliciting questions from patients and reassurance (*n* = 3 each). Other empathy-related behaviours included using a consultation structure, unspecified conversations, more time (*n* = 2 each), discussion of lifestyle issues, checking patient understanding, describing the evolution of the disease, instructing the patient on how to quantify their symptoms, proposing a patient-practitioner partnership, complying with patient wishes and emphasizing comfort and well-being (*n* = 1 each).

**Table 2:** Training Content of Empathy Interventions Extracted Using Content Analysis with Effect Sizes

	Non-specific empathic responses	Explanations of treatment	Reassu-rance	Describe evolution of disease	Instruct patient how to quantify symptoms	Elicit questions from patient	Patient-practitioner partnership	Friendly manner	Non-verbal behaviours (gestures, looking, facial expression)	Consultation structure (appropriate to discussion)	Lifestyle discussion (features outside immediate symptoms)	Checking patient understands	Active listening	Express positive expectation of treatment	More time	Non-specific conversation	Comply with patient wishes	Emphasize comfort and well-being	Total	Effect size (std. mean difference)
Chassany 2006	x	x	x	x	x	x	x												7	− 0.19
Fujimori 2014	x	x	x			x		x	x	x									7	− 0.16
Kaptchuk 2008	x	x						x	x		x	x	x	x	x				9	− 0.52
Little 2015			x					x	x	x			x						5	− 0.35
Soltner 2011		x				x									x	x			4	− 0.02
Vangronsveld 2012	x								x				x						3	− 0.13
White 2012		x						x								x	x	x	5	0.16
	4	5	3	1	1	3	1	4	4	2	1	1	3	1	2	2	1	1		

While the descriptions used consistent terminology for describing components of the training, they were not specific in what that entailed. For example, ‘non-specific empathic responses’ and ‘friendly manner’ could include non-verbal behaviours such as nodding and smiling, or verbal reassurance. Better descriptions of the training components would be required to resolve this.

### Behaviour Change Techniques

Table 3 summarises the BCTs evident from descriptions of the empathy training. Between 1 and 6 (media*n* = 5) BCTs were identified in each empathy training. Due to the scant reporting of training in several of the papers (especially^12–14^), it is likely that the training employed more BCTs, but there was insufficient evidence to code any others.

**Table 3:** Behaviour Change Techniques Used to Train HCPs in Empathy Identified Using the Behaviour Change Technique Taxonomy. Effect Sizes Taken from Howick et al. with effect sizes

	1.1 Goal setting	1.2 Problem solving	1.4 Action planning	2.2 Feedback on behaviour	2.3 Self-monitoring of behaviour	4.1 Instruction on how to perform behaviour	5.3 Information about social/environmental consequences	6.1 Demonstration of behaviour	6.2 Social comparison	7.1 Prompts/cues	6.3 Information about others’ approval	8.1 Behavioural practice	9.1 Credible source	9.2 Pros and cons	Total	Effect size (std. mean difference)
Chassany 2006						x				x			x	x	64	− 0.19
Fujimori 2014		x		x		x		x			x	x	x		67	− 0.16
Kaptchuk 2008				x		x		x				x	x		5	− 0.52
Little 2015	x		x		x	x	x				x				6	− 0.35
Soltner 2011												x			1	− 0.02
Vangronsveld 2012						x							x		2	− 0.13
White 2012									x						1	0.16
Total	1	1	1	2	1	5	1	2	1	1	2	3	4	1	27	

The most common BCT used was *4.1 Instruction on how to perform behaviour* (*n* = 5), followed by *9.1 Credible source* (*n = 4*) and *8.1 Behavioural practice* (*n* = 3). *2.2 Feedback on behaviour*, *6.1 Demonstration of behaviour* and *6.3 Information about others*’ *approval* were evident in two training descriptions. Additionally, Little 2015 employed *1.1 Goal setting*, *1.4 Action planning*, *2.3 Self-monitoring of behaviour* (n.b. this was self-monitoring of a behaviour recorded at baseline, not monitoring of changes in behaviour) and *5.3 Information about social/environmental consequences*; Chassany 2006 used *7.1 Prompts and cues* and *9.2 Pros and cons*; and White 2012 used *6.2 Social comparison*.

### Effect Sizes

The effect sizes for each intervention, as reported by Howick et al. (2018), are shown in Tables 2 and 3. All interventions had a significantly positive effect on psychological outcomes, with the exception of White 2012, which was not statistically significant. Kaptchuk 2008 and Little 2015 demonstrated the greatest effect sizes. These trainings were quite diverse in training methods and BCTs used, with only *Instructions on how to perform behaviour* as a common BCT. Both used self-recording as part of training, though not exclusively to the dataset. The training content overlapped in several areas: they included elements of *Friendly manner*, *Non-verbal behaviours* (*gestures*, *looking*, *facial expression*), and *Active listening*. Due to the diversity of these features, and the paucity of reporting in other interventions, we do not feel that we can draw any strong conclusions from these commonalities. The studies with the highest effect sizes varied greatly in duration (up to 2 h10^17^ versus 20 h^16^), suggesting that empathy training does not need to be long to be effective.

## DISCUSSION

In this paper, we identified the components of HCP training in empathic communication from seven empathy papers based on a recent systematic review,^10^ with no additional papers found from further literature searches and discussions with topic experts. We found that training included a variety of methods that emphasized a spectrum of empathic behaviours and employed a range of BCTs. Face-to-face training, role-playing and videos were commonly used. The most frequent behaviours targeted were providing explanations of treatment, providing non-specific empathetic responses, displaying a friendly manner and using non-verbal behaviours. The most common BCT used to train HCPs was 4.1 Instruction on how to perform behaviour, followed by 9.1 Credible source and 8.1 Behavioural practice. There were some similarities in intervention components between the papers with high effect sizes, but also much diversity: the training methods and content varied greatly. Of particular relevance for the pressured environment of everyday clinical practice, there was little evidence that longer training was beneficial.

To the best of our knowledge, this is the first study to examine in detail the underlying methods and behaviours utilised to train HCPs in delivering empathy. Inadequate theoretical development and consideration of these underlying processes in delivering empathy interventions are likely to have hindered progress in the wider clinical application of empathy interventions. To some extent, this might have contributed to the findings of Howick et al.’s^10^ review in which only small absolute effects were observed. More detailed consideration of these processes is critical and timely in delivering effective and cost-effective empathy interventions. At a time of unprecedented pressures and greater austerity in the UK and other health services, alongside declining patient satisfaction, practitioner empathy could provide a valuable additional tool given the previous evidence of its effect on patient satisfaction, trust, health outcomes and HCP well-being. Our findings highlight key areas that are promising in future development and application towards effective empathy interventions.

Our ability to investigate which empathic behaviours are most effective for improving patient outcomes (Aim 2) was hampered by the lack of reporting adequacy of empathy training methods. Empathy training and empathic behaviours were defined and described in different ways and were reported in varying degrees of detail. For example, Fujimori et al.^15^ provided a detailed schedule of a 2-day workshop, while Soltner et al.^14^ presented a calibration study to check the success of training, the content of which was not described. Furthermore, while some studies did not describe using particular training or behaviour change methods, this does not necessarily mean that such methods were not used. The papers also lacked information on the level of experience the trainers had in education, which could impact on the training’s effectiveness. While we were able to obtain additional information about one intervention by contacting the author (Little et al.^17^), despite attempting contact we were unable to obtain any further details from the authors of the remaining papers. Although there has been recent attention to the better reporting of interventions,^21^ there has been limited work on describing how people are trained to deliver the intervention. This is essential for interventions which deliver a complex behavioural interaction, such as conveying empathy in a clinical consultation. Without a complete description of this, empathy training trials cannot be accurately replicated, or the findings built upon. We recommend a checklist is developed for the reporting of intervention delivery training (perhaps as an extension to the TIDieR framework^21^ for intervention descriptions) where the intervention has a complex behavioural component.

We also found that the seven studies presented in the systematic review were insufficient to draw conclusions about which (combinations of) components of training might have the largest effects on patient health outcomes. Although the studies chosen for Howick et al.’s review^10^ were randomised trials, other reviews and individual trials on empathy may provide additional data (e.g.^22,23^). These studies were excluded from the study because no patient outcomes were reported (e.g. Riess et al. reported patient-rated practitioner empathy but no health outcomes^24^). However, the data from these excluded studies relating to how empathy is trained may be usefully examined to encapsulate current empathy training approaches. Qualitative studies may also be helpful for understanding the broader context and consequences of empathy training.^25^

Another limitation is that the BCT Taxonomy^20^ approach may have been inadequate for encapsulating the BCTs conveyed in the intervention. We found that the interventions applied the same BCTs in different ways—e.g. the 15 min PowerPoint presentation^17^ from a medical student delivered in the workplace to one HCP and the 1-h lecture from an expert to a groups of HCPs^15^ both demonstrated the BCT of “Instruction on how to perform behaviour”, but are not necessarily comparable. This somewhat reductive method erases important distinctions between the interventions. Furthermore, the taxonomy does not permit a BCT to be coded unless it meets strict criteria—therefore, we could not code ‘1.5 Goal Review’ for,^17^ where the taxonomy assumes there must be ‘someone’ delivering the intervention, though self-directed goal reviewing was present.

The heterogeneity in HCPs, training methods and contexts of the studies examined may also contribute to our disparate findings. It is plausible that if a larger set of similar studies had been grouped together, our findings may have elicited different outcomes. Relatedly, the trials in our sample were all English language papers, which may impact upon the generalisability of our findings, and further to this, cultural differences in the HCP-patient relationship may preclude the application of our results in non-western cultures.

This study, like many, is limited by a likely selection bias within the primary studies. It is likely that people who were interested in becoming more empathic chose to participate in these studies. They were motivated to make changes to their practice. It is certainly possible that this effect would be weaker ‘in the wild’, and further work would be needed to explore it. Mitigating this bias, there may have been contamination in the control groups. HCPs in the control groups may have enhanced the way they expressed empathy although they were not trained to do so.

Our study is one of the first to examine the common elements of empathy training for HCPs. Findings suggest that HCPs wanting their practice to reflect current evidence can consider enhancing their friendly manner, empathic responses, non-verbal behaviour and explanations of treatments. However, specific techniques may be more or less appropriate depending on the clinical context. Furthermore, advice to enhance one’s friendly manner, empathic responses and non-verbal behaviour may be too generic to be meaningfully implemented. It is therefore imperative that future studies in this area provide comprehensive, detailed, descriptions of training content and training methods, including the application of any behaviour change techniques. Studies should conform to better intervention reporting standards such as TIDieR^21^ when reporting any training undertaken as part of an intervention, taking care to clearly describe specific behaviours (e.g. nodding) rather than broader categories (e.g. friendly manner). Without this, the studies become impossible to replicate, and it is impossible to extrapolate what aspects of empathy training are effective.

## References

[1] Derksen F, Bensing J, Lagro-Janssen A (2013). Effectiveness of empathy in general practice: a systematic review. Br J Gen Pract.

[2] Howick J, Bizzari V, Dambha-Miller H (2018). Therapeutic empathy: what it is and what it isn't. Journal of the Royal Society of Medicine.

[3] **W. V. Lerberghe**, “The world health report 2008: primary health care: now more than ever.,” World Health Organization, 2008.

[4] NHS, *The NHS Constitution for England,* Department of Health England, 2015.

[5] Neumann M, Edelhäuser F, Tauschel D, Fischer MR, Wirtz M, Woopen C, Haramati A, Scheffer C (2011). Empathy decline and its reasons: a systematic review of studies with medical students and residents. Academic medicine.

[6] **R. Robertson**, **J. Appleby** and **H. Evans**, “Public satisfaction with the NHS and social care in 2016,” *Results and trends from the British social attitudes survey. London: King’s Fund,* 2017.

[7] Griffin SJ, Kinmonth A-L, Veltman MWM, Gillard S, Grant J, Stewart M (2004). Effect on health-related outcomes of interventions to alter the interaction between patients and practitioners: a systematic review of trials. The Annals of Family Medicine.

[8] Dambha-Miller H, Feldman AL, Kinmouth AL, Griffin SJ (2019). Association Between Primary Care Practitioner Empathy and Risk of Cardiovascular Events and All-Cause Mortality Among Patients With Type 2 Diabetes: A Population-Based Prospective Cohort Study. Annals of Family Medicine.

[9] Samra R (2018). Empathy and Burnout in Medicine-Acknowledging Risks and Opportunities. Journal of general internal medicine.

[10] **J. Howick**, **A. Moscrop**, **A. Mebius**, **T. R. Fanshawe**, **G. Lewith**, **F. L. Bishop**, **P. Mistiaen**, **N. W. Roberts**, **E. Dieninytė**, X.-Y. Hu and others, “Effects of empathic and positive communication in healthcare consultations: a systematic review and meta-analysis,” *Journal of the Royal Society of Medicine,* vol. 111, pp. 240-252, 2018. 10.1177/0141076818769477 PMC6047264 29672201

[11] Chassany O, Boureau F, Liard F, Bertin P, Serrie A, Ferran P, Keddad K, Jolivet-Landreau I, Marchand S (2006). Effects of training on general practitioners' management of pain in osteoarthritis: a randomized multicenter study. The Journal of rheumatology.

[12] Vangronsveld KL, Linton SJ (2012). The effect of validating and invalidating communication on satisfaction, pain and affect in nurses suffering from low back pain during a semi-structured interview. European Journal of Pain.

[13] White P, Bishop FL, Prescott P, Scott C, Little P, Lewith G (2012). Practice, practitioner, or placebo? A multifactorial, mixed-methods randomized controlled trial of acupuncture. Pain.

[14] Soltner C, Giquello JA, Monrigal-Martin C, Beydon L (2011). Continuous care and empathic anaesthesiologist attitude in the preoperative period: impact on patient anxiety and satisfaction. British journal of anaesthesia.

[15] Fujimori M, Shirai Y, Asai M, Kubota K, Katsumata N, Uchitomi Y (2014). Effect of communication skills training program for oncologists based on patient preferences for communication when receiving bad news: a randomized controlled trial. Journal of clinical oncology.

[16] **T. J. Kaptchuk**, **J. M. Kelley**, **L. A. Conboy**, **R. B. Davis**, **C. E. Kerr**, **E. E. Jacobson**, **I. Kirsch**, **R. N. Schyner**, **B. H. Nam**, L. T. Nguyen and others, “Components of placebo effect: randomised controlled trial in patients with irritable bowel syndrome,” *Bmj,* vol. 336, pp. 999-1003, 2008. 10.1136/bmj.39524.439618.25 PMC2364862 18390493

[17] Little P, White P, Kelly J, Everitt H, Mercer S (2015). Randomised controlled trial of a brief intervention targeting predominantly non-verbal communication in general practice consultations. Br J Gen Pract.

[18] Dixon-Woods M, Agarwal S, Jones D, Young B, Sutton A (2005). Synthesising qualitative and quantitative evidence: a review of possible methods. Journal of health services research & policy.

[19] Finfgeld-Connett D (2014). Use of content analysis to conduct knowledge-building and theory-generating qualitative systematic reviews. Qualitative Research.

[20] Michie S, Richardson M, Johnston M, Abraham C, Francis J, Hardeman W, Eccles MP, Cane J, Wood CE (2013). The behavior change technique taxonomy (v1) of 93 hierarchically clustered techniques: building an international consensus for the reporting of behavior change interventions. Annals of behavioral medicine.

[21] **T. C. Hoffmann**, **P. P. Glasziou**, **I. Boutron**, **R. Milne**, **R. Perera**, **D. Moher**, **D. G. Altman**, **V. Barbour**, **H. Macdonald**, M. Johnston and others, “Better reporting of interventions: template for intervention description and replication (TIDieR) checklist and guide,” *Bmj,* vol. 348, p. g1687, 2014. 10.1136/bmj.g1687 24609605

[22] Kiosses VN, Karathanos VT, Tatsioni A (2016). Empathy promoting interventions for health professionals: a systematic review of RCTs. Journal of Compassionate Health Care.

[23] Kelm Z, Womer J, Walter JK, Feudtner C (2014). Interventions to cultivate physician empathy: a systematic review. BMC medical education.

[24] Riess H, Kelley JM, Bailey RW, Dunn EJ, Phillips M (2012). Empathy Training for Resident Physicians: A Randomized Controlled Trial of a Neuroscience-Informed Curriculum. J Gen Intern Med.

[25] Krishnasamy C, Ong SY, Loo ME, Thistlethwaite J (2019). How does medical education affect empathy and compassion in medical students? A meta-ethnography: BEME Guide No. 57. Medical Teacher.

